# Building Back Better: Local Health Department Engagement and Integration of Health Promotion into Hurricane Harvey Recovery Planning and Implementation

**DOI:** 10.3390/ijerph16030299

**Published:** 2019-01-23

**Authors:** Mallory Kennedy, Shannon Gonick, Hendrika Meischke, Janelle Rios, Nicole A. Errett

**Affiliations:** 1 The University of Washington School of Public Health Department of Health Services, Seattle, WA 98195, USA; hendrika@uw.edu; 2 The University of Washington School of Public Health Department of Environmental and Occupational Health Sciences, Seattle, WA 98195, USA; shannongonick@gmail.com (S.G.); nerrett@uw.edu (N.A.E.); 3 The University of Texas Health Science Center (UTHealth) School of Public Health, Houston, TX 77030, USA; Janelle.Rios@uth.tmc.edu

**Keywords:** disaster recovery, public health, resilience

## Abstract

Disaster recovery provides an opportunity to build healthier and more resilient communities. However, opportunities and challenges encountered by local health departments (LHDs) when integrating health considerations into recovery have yet to be explored. Following Hurricane Harvey, 17 local health and emergency management officials from 10 agencies in impacted Texas, USA jurisdictions were interviewed to describe the types and level of LHD engagement in disaster recovery planning and implementation and the extent to which communities leveraged recovery to build healthier, more resilient communities. Interviews were conducted between December 2017 and January 2018 and focused on if and how their communities were incorporating public health considerations into the visioning, planning, implementation, and assessment phases of disaster recovery. Using a combined inductive and deductive approach, we thematically analyzed interview notes and/or transcripts. LHDs reported varied levels of engagement and participation in activities to support their community’s recovery. However, we found that LHDs rarely articulated or informed decision makers about the health impacts of recovery activities undertaken by other sectors. LHDs would benefit from additional resources, support, and technical assistance designed to facilitate working across sectors and building resilience during recovery.

## 1. Introduction

In 2017, the United States was hit by a historic number of billion-dollar disasters that cost more than $300 billion and claimed 362 lives [1]. As our climate continues to change, certain types of extreme weather events are expected to increase in frequency and intensity in the future [2,3]. 

Given these harsh realities, it is imperative that communities are able to minimize the impacts of and cope with future disasters—that is, they must become resilient. While various definitions for resilience exist [4], Chandra et al. define it as: “the ongoing and developing capacity of the community to account for its vulnerabilities and develop capabilities that aid that community in 1) preventing, withstanding, and mitigating the stress of a health incident; 2) recovering in a way that restores the community to a state of self-sufficiency and at least the same level of health and social functioning after a health incident; and 3) using knowledge from a past response to strengthen the community’s ability to withstand the next health incident” [5]. Resilience necessitates that communities build robust physical, social, and economic infrastructure and invest in population health, education, and natural environments that offer protection during and after disasters [6]. 

In recent years, federal agencies have increasingly recognized the importance of community resilience; some have incorporated it into their disaster-related frameworks and grants [7]. For example, the Centers for Disease Control and Prevention (CDC) includes community resilience as one of six domains for their Preparedness Capabilities, and the first strategic objective of the National Health Security Strategy is to “build and sustain healthy, resilient communities” [8,9].

Disaster recovery provides an opportune time to build resilience due to the influx of resources into a community and the unique chance to invest in and rebuild society for the long-term—to “build back better” [10,11]. In fact, the Federal Emergency Management Agency (FEMA) states in the “purpose” section of its National Disaster Recovery Framework that it aims to reduce vulnerabilities and build resilience through recovery [12].

Although there have been several examples of communities building resilience through recovery, including after Hurricane Katrina and Superstorm Sandy [11], barriers remain [13,14,15]. Such barriers include the conflict between the desire to reflexively return to pre-disaster conditions and the desire to rebuild in a way that is attentive to equity and health [13,14]. This creates tension between “snapback (essentially restoration of the past) and redevelopment that addresses future needs and vision” [13]. During recovery from previous disasters, communities have largely attempted to build resilience by adopting infrastructure-centric approaches rather than those that focus on people, equity, and health [10]. Furthermore, the disaster recovery process is subject to time compression wherein—in some cases, depending on the disaster—an entire community must be redeveloped and rebuilt, services must be restored, and livelihoods must be returned [15]. Time constraints present a significant challenge for building back better, as the process of community development is complex even under ideal circumstances. These barriers have the potential to constitute significant missed opportunities for building back communities in ways that are more conducive to resilience—ones that communities may no longer afford to forego with anticipated increases in disaster frequency and intensity.

The 2015 Institute of Medicine (now the National Academy of Medicine (NAM)) report entitled, “Healthy, Resilient, and Sustainable Communities After Disasters” (hereafter, “the NAM report”), highlighted these barriers and proposed a framework for communities to build health resilience through recovery. The report proposed the use of a health-in-all-policies (HiAP) approach to community recovery to exploit the natural synergies between recovery and resilience building processes [10]. The National Association of County and City Health Officials (NACCHO) describes HiAP as an approach to governmental decision-making that “ensure[s] that policy decisions have neutral or beneficial impacts on health determinants. HiAP emphasizes the need to collaborate across sectors and break down “silos” to achieve common health goals.” [16]. For example, in the context of recovery, an HiAP approach might include a community including a grocery store or behavioral health clinic on a public transportation route that has been modified to accommodate displaced populations or inaccessible areas, or undertaking efforts to increase access to parks or sidewalks to promote physical activity. NACCHO describes local health departments (LHDs) as “natural leaders to implement HiAP at the local level” [16]. The NAM report similarly recommends that LHDs be engaged in community-level, multiagency recovery planning and implementation to ensure that recovery activities, such as rebuilding housing and community infrastructure, strategically and proactively address social determinants of health and build resilience, equity, and sustainability, and lead efforts to restore healthcare and public health systems to achieve long-term community health goals [10]. Table 1 outlines the strategic planning phases of disaster recovery described by the NAM report.

Despite the detailed approach outlined in the NAM report and the fact that HiAP has been promulgated by researchers and practitioners alike as a tool for addressing social determinants of health and promoting health equity through decision-making [17], there remains little evidence of if and how LHDs are leading their communities to systematically use an HiAP approach to recovery or how LHD recovery planning and implementation aims to meet long-term community health goals. To date, research has focused on the role of community-based organizations and faith-based organizations as LHD partners in recovery [18,19], but studies have not yet explored the facilitators and barriers LHDs face to building resilience during recovery and the extent to which LHDs are working across sectors to implement an HiAP approach to recovery.

Hurricane Harvey pummeled Southeast Texas from August 25–31, 2017 [20]. The hurricane pelted the areas in its path with rainfall of between 4.62 and 56 inches, resulting in a cumulative 33 trillion gallons of water [21,22]. Harvey displaced more than 30,000 people and wreaked havoc on more than 200,000 homes and businesses, with an estimated cost of $125 billion—a price tag second only to Hurricane Katrina’s [23,24]. In light of these devastating impacts, Hurricane Harvey was recognized as an opportunity to strategically and proactively rebuild communities to be healthier and more resilient [11,25].

In response, we sought to explore: the extent to which LHDs participated in Hurricane Harvey disaster recovery planning and implementation; LHDs’ completed short-term recovery activities and plans for long-term recovery activities; and the extent to which such recovery processes were being leveraged to build healthier, more resilient communities.

## 2. Materials and Methods

Semistructured interviews with LHD and/or Office of Emergency Management officials were conducted in December 2017–January 2018 using an interview guide informed by the NAM report and intended to address the ways in which health considerations were integrated into the core elements of the recovery strategic planning framework presented in the report. For example, we asked questions such as: “are there any recovery activities that are being led by non-health sectors that might have public health impacts?” and “have you had the opportunity to inform these efforts being led by non-health sectors or to provide them with guidance, information, or input about how those activities might impact public health?” We relied on respondents to self-identify their agency’s recovery activities, emphasizing that the intent of the interview was to focus on activities that followed their agency’s response as needed.

Three members of the research team (MK, SG, and NE) developed the interview guide and two members (JR and HM) reviewed the guide. Five additional public health practitioners and academics reviewed and provided feedback on the guide. Reviewers had backgrounds and expertise in disaster recovery, emergency preparedness, local and state public health practice, and/or qualitative research. On November 3, 2017, the University of Washington Human Subjects Division (HSD) determined that the study was human subjects research that qualified for exempt status (category 2).

The study team first identified all of the counties eligible for individual assistance as part of FEMA’s Texas Hurricane Harvey Disaster Declaration (DR-4332). We then used the Texas Department of State Health Services’ website to identify local public health organizations and contacts in these counties. Counties for which there was no listed local public health organization or contact were excluded. We recruited 26 agencies in total via email or telephone to participate in an interview. In some cases, the county public health officials directed us to other individuals (e.g., preparedness coordinators) to participate in the study. Of the 26 agencies that were contacted, 10 agreed to participate in an interview. Nonresponding agencies either: failed to respond to multiple contact attempts; expressed interest in participating in an interview but were unresponsive to subsequent attempts to schedule an interview; indicated that their agency was not involved in recovery activities; or felt they were not the appropriate person to participate in the interview and failed to identify an appropriate alternate to participate in the study.

We conducted semistructured interviews with 17 respondents from 10 different organizations—nine LHDs and one Office of Emergency Management. In some cases, the individual initially contacted for an interview requested that one or more additional individuals be included in the interviews due to their ability to speak to different aspects of their agency’s recovery work. Table 2 presents the job titles of interviewees.

Respondents were in their roles for time periods between less than one year and more than 10 years. They represented areas with: populations ranging from less than 50,000 to greater than 500,000; the population living below the poverty level ranging from less than 10 percent to approximately 20 percent; the population speaking a language other than English at home ranging from less than 5 percent to greater than 40 percent; and the mean household income ranging from approximately $60,000 to $100,000 [26,27,28].

Interviews were conducted in person or by phone after participants provided informed consent to participate, and all but one interview was audio-recorded, transcribed, coded, and analyzed. For the interview where the interviewee declined to be recorded, detailed notes taken during the interview were coded and analyzed in lieu of transcribed text.

We used a combined inductive and deductive approach to conduct a thematic analysis of the data. First, we developed a priori codes based on the research question and the NAM report framework. Next, we reviewed all of the transcripts, noting additional themes that warranted inclusion as codes. We then drafted a preliminary codebook. Two team members co-coded one transcript, discussed and adjudicated the results, and refined the codebook accordingly. Finally, one team member coded and analyzed the remaining interviews in NVivo for Mac v11.4.3 (QSR International, Doncaster, Victoria, Australia). Themes that emerged across respondents were summarized. The level of analysis for this study was the agency rather than the individual interviewee. Throughout this paper, “respondent” refers to the responding agency.

## 3. Results

The NAM report proposed a four-phase approach for recovery planning similar to that used in strategic planning: developing a vision of a healthy community to guide recovery; using assessments to inform recovery planning; planning for recovery; and implementing recovery activities [10]. Herein, we describe LHD engagement and health integration into each of these phases.

### 3.1. Visioning Recovery for Resilience

The majority of respondents did not report participation in the development or implementation of a community-wide vision to develop a healthier, more resilient, and sustainable community following Hurricane Harvey. Rather than striving to create a healthy community, respondents most frequently described the chief objective of their recovery processes as returning their community to normal. Respondents noted that recovery discussions revolved around “trying to stabilize” the community, and “restoring (infrastructure) to pre-disaster condition.” 

In general, respondents described reactive approaches to recovery. Respondents’ recovery processes focused on providing resources and addressing community needs as they were identified, rather than setting community recovery goals and acting to achieve them. Even when respondents anticipated community needs as opposed to reacting to them, their efforts largely focused on hardening critical infrastructure or preventing future short-term health impacts from the storm (e.g., flu and mold prevention), rather than aiming to improve long-term community health. 

One respondent did describe attempting to build resilience from future disasters through their recovery efforts, and stated that the goal of their community’s long-term recovery group “is to not only address the needs of this particular disaster, but also, in some way, shape, or form, be able to look at future disasters, and see, at least, if we can put some things in place that might be beneficial.”

### 3.2. Planning

Pre-Event Planning. All respondents described engaging in pre-event planning. Planning activities most commonly discussed were emergency preparedness exercises, drills, and trainings. Most of these activities focused on components of the response and short-term recovery, such as trainings on the incident command system (ICS), shelter plan drills, and mass vaccination. Respondents also reported engaging in other planning efforts—such as developing written plans or participating in less formalized planning efforts—which also largely focused on response and short-term recovery (e.g. mass care, evacuation, mass vaccination, shelter operations, ICS operations, debris removal, and post-event disease surveillance). 

Only two respondents described developing plans that focused on intermediate and long-term recovery. As one respondent remarked, “I don’t know how much the plan has been set up to guide the recovery effort, because it’s really a response plan. It’s what are you doing in those immediate 24 hours to a week, 2 weeks out after the event…that’s kind of where the plan begins and ends.” 

Post-Event Planning. Only half of the respondents reported having a formalized structure or group responsible for post-event recovery planning (e.g., long-term recovery groups; a “virtual ICS” structure). Respondents without formalized structures for recovery decision-making reported simply responding to community needs as they were identified, i.e., without any intentional planning activities.

The respondents that reported having formalized structures for post-disaster recovery planning described an approach to recovery that included more formalized processes for carrying out recovery activities, communicating with partners, and working with partners to address recovery issues, than did respondents without formalized structures. While most of these respondents reported engaging partners in recovery decision-making processes, sharing resources across organizations, and undertaking a more whole-community approach to recovery, in some cases, their post-event planning processes were fragmented. For instance, respondents reported having long-term recovery groups with different subcommittees but did not always indicate whether or how subcommittees looked to exploit natural synergies across their work.

Despite these formal recovery planning structures, none of the respondents reported developing written plans post-event to guide recovery or using preexisting community-wide plans—such as community-level strategic plans—to guide community recovery efforts. Instead, recovery planning was driven largely by community needs articulated by a variety of sources, including elected officials, social service professionals working in the community, or residents. 

LHD Role in Recovery. According to the NAM report, the public health sector has a large role in building resilience through recovery. For example, LHDs may provide data and information to other sectors to ensure they consider the long-term health impacts of their decisions and actions [10]. To play such a role, LHDs must have meaningful engagement in recovery planning and decision-making. To this end, we asked LHD respondents to describe the role of their agency in recovery and the extent to which recovery prioritizes other areas over public health. 

Most LHD respondents reported being involved in their communities’ recovery decision-making processes to some extent. Several LHD respondents provided recommendations and input to facilitate decision-making, such as decisions around aerial mosquito spraying. Several others reported influencing the direction of recovery in other ways. For instance, one LHD helped to develop the community’s long-term recovery group and sits on the group’s executive committee. From that position, they are able to influence the direction of recovery, including subgroups in which the LHD does not participate directly. Other LHDs said that their local government looks to them to make decisions, that they convey their community’s most pressing needs directly to state representatives and request resources, and that they assume leadership roles in whole-community recovery efforts, such as leading neighborhood restoration centers.

While most respondents reported having a “seat at the table”, the extent to which their participation was meaningful and influential varied. One respondent remarked that despite the LHD’s involvement in decision-making bodies, they struggle to gain support for their recommendations. Several LHDs’ participation in the recovery decision-making processes consisted mainly of participating in health-specific decision-making processes and bodies or updating other stakeholders on their recovery activities and processes rather than influencing the direction of recovery. 

Respondents reported collaboration with a variety of stakeholders on elements of response and recovery planning and implementation (Table 3). 

### 3.3. Implementation

We found minimal evidence of LHDs leveraging the recovery process to meet pre-disaster goals. Only one respondent reported tying recovery activities to their LHD’s strategic plan objectives, noting that in tandem with recovery efforts, they were encouraging healthy habits and promoting other population health initiatives. 

Additionally, most respondents did not describe any actions being undertaken—either by the LHD or by partner organizations—to mitigate their community’s extant health issues through recovery. The only example of a community potentially mitigating health issues through recovery was through updating building codes to improve the community’s housing. As Harvey exposed substandard housing in the respondent’s community, they indicated that their county’s decision-makers (e.g., county judges, elected officials) were debating whether to adopt and enforce stricter building codes to ensure homes are rebuilt to a higher standard.

Respondents’ public health- and healthcare-related recovery activities focused on restoring and delivering essential public health and healthcare services. The most commonly described intermediate/long-term recovery activity related to these areas—reported by over half of the respondents—was health and safety inspections (e.g., at restaurants and schools). Several respondents described their efforts to restore potable water and sanitation, including testing water samples, providing input on when to lift their community’s boil water advisory, and restoring sanitation services. Several respondents also reported that community-based organizations were providing health services to residents, including services targeted to low-income individuals, older adults, and the “medically vulnerable”. Additionally, several respondents reported conducting vector control, disease surveillance, and outbreak investigations, and administering flu and tetanus vaccines. 

Nearly all respondents described communication challenges with partners and the public. Respondents cited staff turnover and a lack of communication and longstanding relationships with their healthcare (e.g., dialysis centers, pharmacies) and non-healthcare partners as the cause of challenges during and post-Harvey. Due to these challenges, respondents identified a need to: strengthen their relationships and communication with all partners pre-disaster; more clearly delineate roles and responsibilities with partner organizations; and identify the resources to be provided by each organization during and post-disaster. 

Nearly all respondents encountered resource-related challenges in recovery planning and implementation. Respondents cited small tax bases, chronic underfunding of public health, and lack or delay of volunteers and resources as undermining their abilities to carry out all of their recovery activities. Respondents identified the need to streamline resource request processes and pre-identify partner organizations able to furnish resources and support in a disaster.

### 3.4. Assessments

The most commonly reported mechanism for assessing community status post-disaster was through the collection of anecdotal and informal information about community needs. These informal assessments were reported by more than half of the respondents. Nearly half the respondents reported conducting or using data gathered through formalized needs assessments, including conducting a post-Harvey community assessment for public health emergency response (CASPER) or other needs assessment or relying on CASPER data collected after a previous disaster. However, respondents did not describe conducting follow-up formalized assessments to identify ongoing community needs or to evaluate recovery efforts. Several respondents also indicated that their recovery monitoring included surveillance of health outcomes that could be related to Harvey, such as respiratory illnesses related to mold exposure and infectious diseases. Only one LHD reported conducting formal pre- and post- disaster community health assessments.

Several respondents reported that their community conducted damage assessments, with some public health relevance (e.g., academic institutions used drones to observe flooding and damage patterns, allowing for the LHD to monitor hospital repairs). Finally, a few respondents reported tracking recovery progress through their structured recovery groups and partners (e.g., “report-out” meetings with their partners in which partners described their progress). 

## 4. Discussion

Our findings suggest respondents face barriers to building resilience during disaster recovery and that thought leadership around health resilience building in disaster recovery may not be translating to practice [10,11,25]. 

Despite emphasis on the importance of adopting a vision of a healthy, resilient community to guide recovery efforts, the majority of LHDs we interviewed reported implementation of reactive recovery strategies focused on returning the community to “normal” and did not pursue activities designed to meet preexisting goals, mitigate health issues, or build resilience in the recovery activities that they had performed to date. 

Our research highlights challenges faced by LHDs while planning and training for long-term recovery. Public health practitioners need accessible tools, resources, and technical assistance designed to facilitate the role of LHDs as leaders in long-term recovery and champions of HiAP and building back better. A carefully considered plan developed in conjunction with community partners can help minimize a community’s reflex to focus on immediate and short-term needs. LHDs need tools to help them to advocate for their inclusion and meaningful participation in recovery planning groups and demonstrate their value in an environment with so many competing interests. LHDs must also be made aware of successful examples of interdisciplinary recovery structures designed to facilitate an HiAP approach, minimize the duplication of recovery efforts, and combine resources for a community-wide, synergistic effect focused on long-term resilience building. We recommend that efforts to increase awareness of extant recovery planning tools (e.g., FEMA’s Pre-Disaster Recovery Planning Guide for Local Governments) be undertaken, and that communities with robust recovery plans developed through interdisciplinary efforts share broadly their plans and strategies for inclusive planning. Brief planning documents or templates could help to distill extant recovery planning documents into more digestible formats, and trainings on planning guidance and strategies could also be developed to assist LHDs in developing recovery plans.

Our findings indicate that LHDs are missing opportunities to conduct, or use data from, assessments to monitor recovery progress and evaluate the impact and effectiveness of recovery strategies on community health and well-being [10]. This lack of a data-driven recovery may be closely tied to the resource and staff capacity challenges reported by nearly all respondents. There is a potential for LHDs to leverage their epidemiology divisions and relationships with academic partners, as these entities may possess methodological expertise and have an understanding of baseline community health conditions to facilitate the collection, analysis, and monitoring of baseline and recovery-related community health data. LHDs would also benefit from additional technical assistance and tools designed to build disaster data collection and analysis capacities.

Different disasters yield different levels of damage and provide different opportunities for recovery and reconstruction. Additional research is necessary to assess the extent to which different types of hazards and disasters are more or less likely to prompt communities to incorporate public health considerations into recovery. Similarly, future research should look beyond Texas for examples of building back better, as the Texas public health structure is unique and might yield different implications for building back better. Moreover, the design of this study (e.g., collecting perspectives from one type of community agency) did not enable us to gather a comprehensive picture of the recovery efforts underway in each community. In addition, it is possible that nonresponding agencies were systematically different than those that participated in our study. We also conducted interviews approximately three months post-Harvey, at a time when recovery was still ongoing in some communities, and were thus unable to capture the breadth and depth of all of the long-term recovery efforts for the communities we interviewed. Additional research designed to capture perspectives from individuals working on longer-term Hurricane Harvey recovery and with individuals outside of the health sector to assess the extent to which health considerations are reflected in recovery goals or activities should be conducted. 

## 5. Conclusions

The findings from this study provide valuable insight into the extent to which Southeast Texas-based LHDs that were affected by Hurricane Harvey are meaningfully engaged in cross-sectoral disaster recovery, and the extent to which initial Hurricane Harvey recovery activities were attentive to health and resilience. The study illuminates the barriers and facilitators to these LHDs’ ability to implement an HiAP approach to recovery and leverage recovery resources to build healthy, sustainable, and resilient communities. Results can inform strategies used by emergency and community planners and responders to better coordinate a community-wide disaster recovery that is forward-thinking and enhances community resilience in the long term. 

The opportunity to build healthier, more resilient communities through disaster recovery has been well described. However, the findings from this study suggest that LHDs may lack the resources, strategies, tools, and other supports necessitated by an HiAP approach to recovery. Without these supports, it is likely that our communities will continue to miss opportunities to improve long-term health and improve our ability to mitigate and cope with future disasters. 

## Figures and Tables

**Table 1:** Strategic planning phases of disaster recovery, adapted from the NAM report [10].

Phase	Characteristics
Visioning	Community engages stakeholders via a “broad-based”, inclusive process to develop a vision of a healthy community that can be incorporated into disaster-related planning and implementation [10] Vision aims to bolster community health, resilience, and sustainability Vision aims to reduce health inequities
Assessment	Assessment provides insight into the community’s “current status and desired state” in terms of health, hazard vulnerability, and other resilience-related factors Assessment results are shared widely across community stakeholders Assessments inform disaster recovery priorities, plans, and activities through a “continuous feedback loop” of assessment, planning, and implementation
Planning	Diverse sectors and community stakeholders work together to incorporate health considerations into planning activities Actors from the public health sector participate purposefully in recovery planning Actors from the public health sector articulate to other sectors the “potential health impacts of recovery decisions” and activities
Implementation	Community stakeholders and actors employ a “collaborative approach” to recovery that is anchored in the vision of a healthy community Implementation promotes the sharing of resources, information, and data Recovery activities aim to improve long-term health and build resilience Assessments of implementation inform subsequent recovery activities and decisions

**Table 2:** Number of respondents by position.

Number of Respondents	Positions
5	Health Department Director
4	Public Health Emergency Preparedness Coordinator
2	Other Health Department Leadership
1	Emergency Management Coordinator
1	Epidemiologist
1	Public Health Nurse
1	Staff Analyst
1	Communications Staff
1	Purchasing/Finance Staff

**Table 3:** Key collaborators and activities.

Partner Organization(s)	Activities
Emergency management	response planning, including shelter plan drills; response implementation, including coordinating mass vaccination clinic; communicating with the public; creating long-term recovery group
Other local government agencies (e.g., law enforcement, mosquito control, Roads and Bridges, county mental health agencies, environmental health, flood agencies, social services, purchasing)	response planning, including developing and sharing list of individuals with functional and access needs and evacuation planning; response implementation, including triaging patients; hotwashes and after-action reviews; recovery planning; recovery implementation, including vector control, providing mental health support to community members, disseminating mental health-related messages, providing data to inform public health recovery purchasing, identifying community needs; resource allocation
Local decision-makers (e.g., judge, Commissioner’s Court, elected officials)	providing resources; response planning; response implementation; recovery planning and decision-making, including around vector control; recovery implementation; identifying community needs
State and federal partners	response implementation, including evacuation; resource allocation; discussing recovery-related challenges; conducting hotwashes and after-action workshops; sharing information
Schools and academic institutions	response planning, including identifying individuals with functional and access needs; response implementation, including mass vaccination clinics; assessment, including assisting with cot-to-cot surveys in shelters
Community Recovery Group (comprised of private sector, local nonprofits, governmental entities, faith-based organizations, schools, and other entities)	identifying community needs; recovery planning, including attempts to minimize recovery activity duplication; sharing information and resources; hosting community meetings; recovery implementation, including mental health counseling; assessment, including home assessments
Foundations, nonprofits, community-based organizations, and non-governmental organizations (e.g., American Red Cross, United Way, Medical Reserve Corps, legal aid organizations, workforce development programs)	response implementation, including shelter operations; serving as fiduciary agent for recovery group; providing recovery funding; recovery planning, including coordinating recovery collaborative group; recovery implementation, including mold remediation, mold education and outreach, alerting the community of contractor fraud, hosting a pop-up shop for basic needs, case management, providing mental health services, providing home cleanout kits and improvement tools; conducting Community Assessment for Public Health Emergency Response (CASPER)
Healthcare partners (e.g., nursing homes, dialysis centers, hospitals; healthcare coalitions)	response planning, including assessing healthcare system readiness and ensuring healthcare facilities remain open during the disaster; response implementation, including evacuation; providing funding for health-related supplies and equipment
